# A Case of Ovarian Dysplasia and a Vaginal Fibroleiomyoma in a Young Golden Retriever

**DOI:** 10.3389/fvets.2021.793186

**Published:** 2021-12-24

**Authors:** Samantha McCarter, D. Phillip Sponenberg, Geoffrey Saunders, Julie Cecere

**Affiliations:** ^1^Virginia-Maryland College of Veterinary Medicine, Blacksburg, VA, United States; ^2^Department of Biomedical Sciences and Pathobiology, Virginia-Maryland College of Veterinary Medicine, Blacksburg, VA, United States; ^3^Department of Small Animal Clinical Sciences, Virginia-Maryland College of Veterinary Medicine, Blacksburg, VA, United States

**Keywords:** ovarian dysplasia, vaginal fibroleiomyoma, persistent estrus, hyperplastic granulosa cells, disorder of sexual development, dog

## Abstract

This case demonstrates a unique ovarian congenital anomaly that likely contributed to the development of a rare fibroleiomyoma in the cranial vagina of a young bitch. A 13 month old intact female Golden Retriever presented to the veterinary teaching hospital for urinary incontinence, hematuria, and persistent vaginal discharge. Physical examination revealed a mucopurulent serosanguinous malodorous vulvar discharge, and after further diagnostics was reclassified as persistent estrus. Abdominal palpation and ultrasound revealed uterine thickening and poorly visualized ovaries. The reproductive tract was removed during an ovariohysterectomy, revealing small ovaries and a white anterior vaginal mass. Histopathology revealed dysplastic ovaries with hyperplastic granulosa cells and a benign vaginal fibroleiomyoma. These morphologic changes are consistent with elevated estrogen levels. It was thus concluded that her persistent estrus and the fibroleiomyoma were both secondary to persistent estrogen production by the hyperplastic granulosa cells.

## Background

Various ovarian conditions relate to disrupted ovarian development or to age-related changes. Ovarian hypoplasia is infrequent and is associated with a deficiency of germ cells. Hypoplastic ovaries are dysfunctional and typically secrete low amounts of estrogen due to an absence of granulosa cells that correlates to the absence of germ cells (1). In normal ovaries, proliferation of the sex-stromal cells, including granulosa cells, typically increases with age. While hyperplasia of these structures can occur, it is typically in geriatric bitches that have experienced multiple estrous cycles and is rare in juvenile animals (1). The proliferation of granulosa cells leads to a significant amount of estrogen release, even in cases without follicles present.

Sex steroid hormones play a significant role in the initiation, promotion, and progression of the carcinogenesis cascade (2). The effects on smooth muscle of the genital tract, can lead to leiomyomas, which are benign neoplasms of these cells. A 2013 study showed estrogen receptor-α and progesterone receptors were expressed in 56.3 and 84.4% of canine leiomyomas, respectively (3). The presence of these receptors indicates that estrogen and progesterone frequently play a role in the development of leiomyomas. Increased levels of these hormones can accelerate the growth of genital tumors.

This present case demonstrates the unusual circumstances that ovarian dysplasia, in synchrony with granulosa cell hyperplasia, can lead to genital neoplasia in a juvenile animal under specific conditions, and can present clinically as persistent estrus.

## Case Report

A 13 month old intact female Golden Retriever bitch was presented to the Virginia Maryland College of Veterinary Medicine's Teaching Hospital with perceived urinary incontinence and a persistent estrus. She weighed 21.6 kg and had a BCS of 4/9. The patient had a history of hematuria and prolonged estrus of ~4 months duration, confirmed via serial observation of superficial epithelial cells on vaginal cytology. The patient was treated for urinary incontinence and multiple urinary tract infections without resolution. Four days before admission, *Enterococcus* spp. growth on a urine culture was documented and the patient began treatment with sensitivity-selected antibiotic therapy.

Physical examination revealed a mucopurulent serosanguinous malodorous vulvar discharge. Significant diagnostic findings included a low (0.42 ng/mL) serum progesterone, vaginal cytology consisting of superficial epithelial cells without significant numbers of neutrophils, no growth on urine culture collected by cystocentesis, and urine sediment that lacked evidence of inflammation. There was no notable mammary gland development.

**Figure 1:**
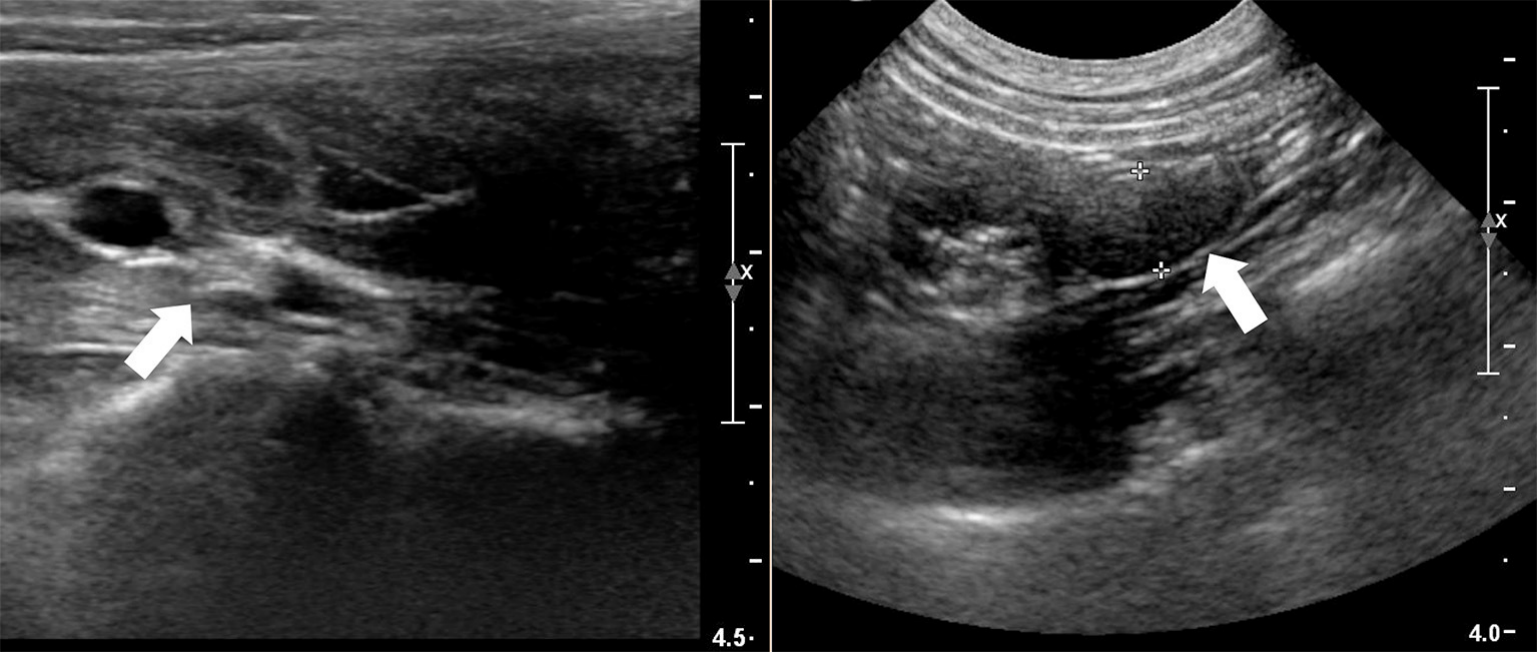
Ultrasound imaging of the cervical thickening at the mass (left arrow) and of the poorly visualized left ovary (right arrow).

Abdominal palpation suggested a thickened uterus; ultrasound examination confirmed this finding, along with a small amount of fluid that was present in the uterus. Additionally, a thickened cervix and poorly visualized ovaries were found (Figure 1). Abdominal ultrasound of the urinary tract revealed moderate bilateral pyelectasis, with no hydroureter visualized. The absence of clinical polyuria along with a history of urinary tract infections suggested that these findings likely indicated pyelonephritis rather than other changes in the urinary tract.

Accompanying the reproductive and urinary abnormalities was moderate bilateral medial iliac lymphadenomegaly, which is most likely consistent with a reactive or inflammatory response. These results, with the history, were consistent with prolonged estrogen influence. The urinary incontinence was redefined as vulvar discharge attributed to uterine secretions secondary to estrogen stimulation.

An elective ovariohysterectomy was performed. Poor ovarian development was noted, along with a firm white cervical mass that extended into the cranial vagina.

**Figure 2:**
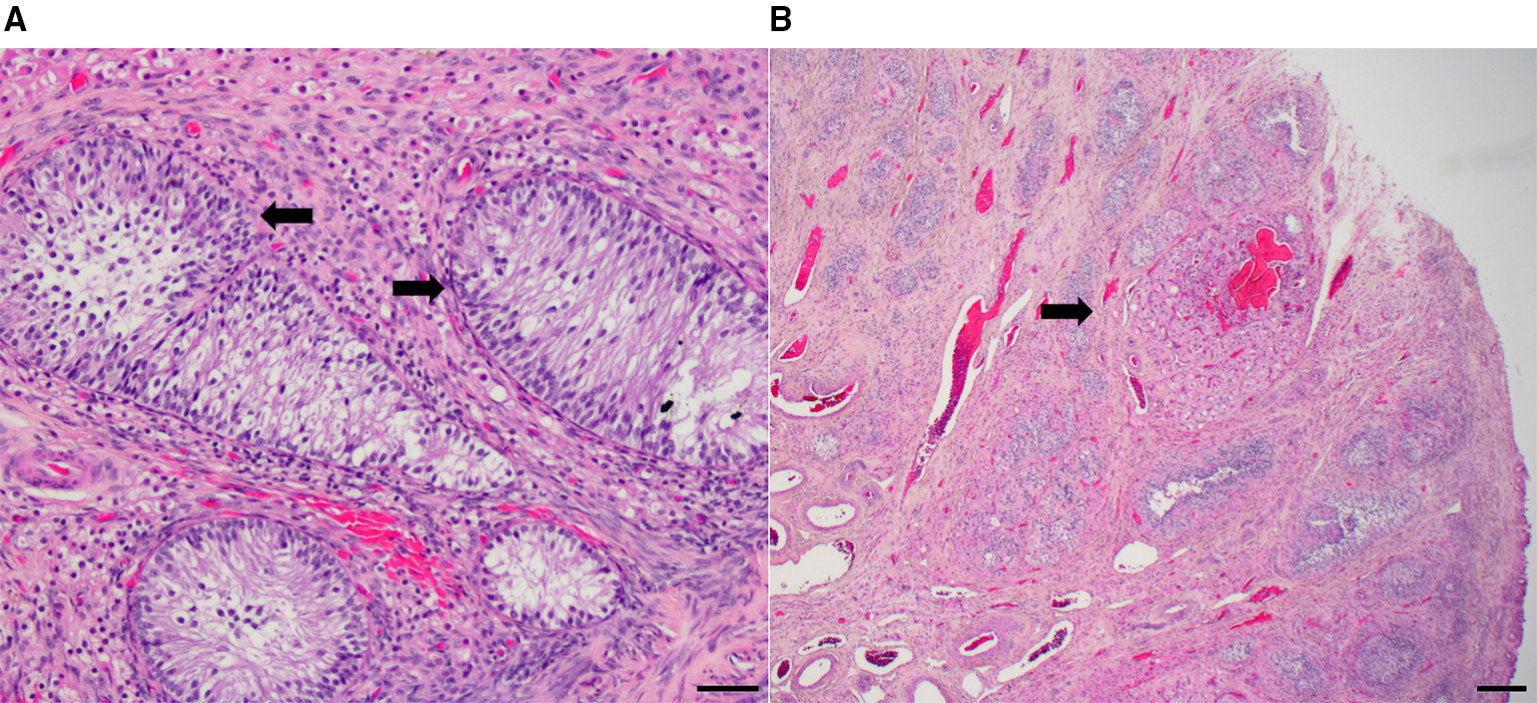
**(A)** Ovary with granulosa cell hyperplasia (arrows) (H&E stain; bar = 20 microns). **(B)** A single corpus luteum (arrow) (H&E stain, bar = 200 microns).

The ovaries, uterus, and anterior vagina were submitted for histopathology, which documented normal uterine morphology (7–9 mm diameter) under estrogen stimulation. The ovaries were small (7 × 8 × 8 mm), and no oocytes or follicles were present. Sex cord stromal cells were prominent, large, and had vacuolated cytoplasm. A single corpus luteum was present (Figure 2). The hyperplastic sex cord stromal cells are granulosa cells and these were suspected to be responsible for persistent estrogen production.

**Figure 3:**
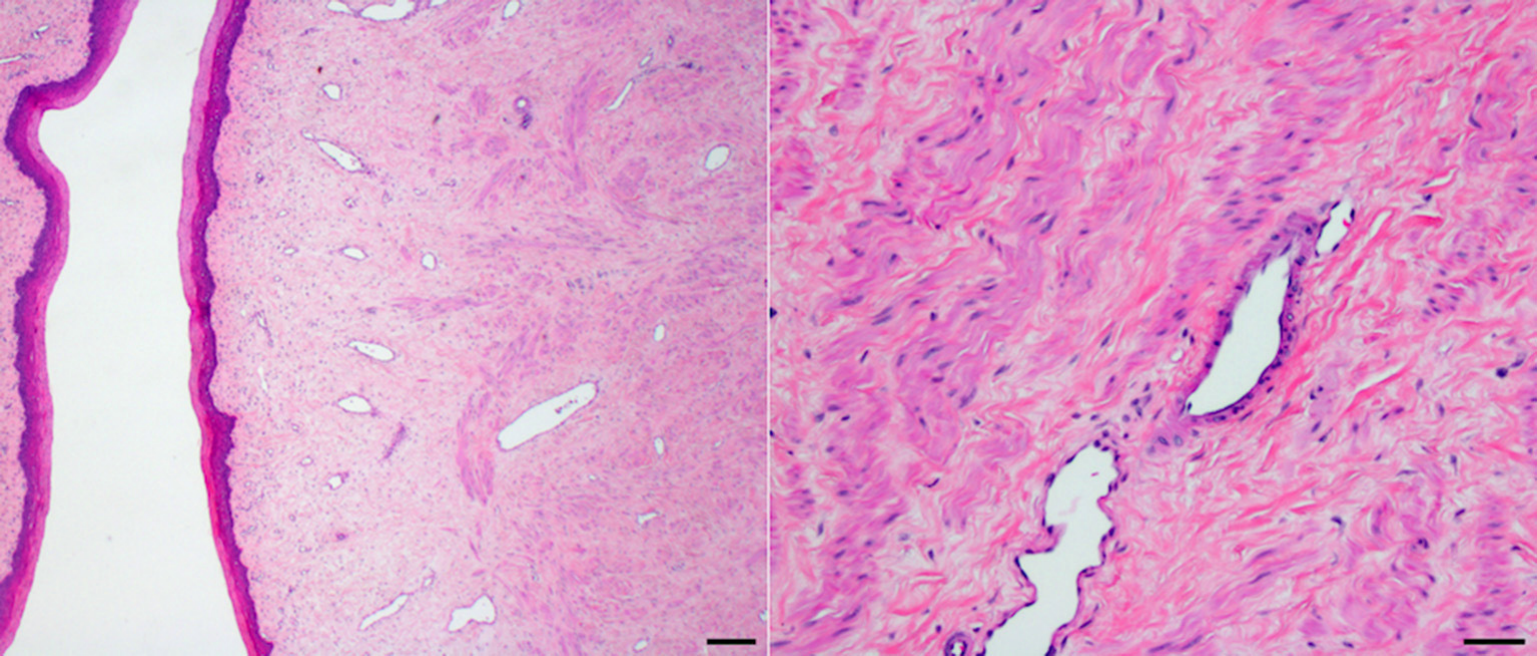
Vaginal fibroleiomyoma (H&E stain; bar = 200 microns and 20 microns).

A benign vaginal fibroleiomyoma (10 × 10 × 15 mm) was present in the anterior vagina adjacent to the cervix (Figure 3). The mass was encapsulated and formed of very elongated cells with abundant uniformly-staining eosinophilic cytoplasm. These cells were arranged in interlacing bundles in a scant fibrous stroma. The nuclei were pale-staining, long oval, and blunt-ended. Most nuclei contained a single prominent nucleolus. No mitoses were observed. A sample of the mass stained positive for smooth muscle actin.

The patient made a full recovery after the ovariohysterectomy.

## Discussion

Ovarian hypoplasia secondary to germ cell deficiency is an infrequent ovarian developmental anomaly, and hypoplastic ovaries usually secrete only low amounts of estrogen (1). A non-follicular source of estrogen is sex-stromal cells, including granulosa cells, which can proliferate with increasing age. Hyperplasia of the sex stromal cells can occur, but it is typically in geriatric bitches and not in juvenile animals (1).

Estrogen synthesis predominantly occurs within granulosa cells using androgens provided by the theca cells (4). Even in the absence of follicles, it has been documented that the proliferation of granulosa cells in sex cords can lead to a significant amount of inhibin release, which can suppress FSH levels (5). The excessive production of estrogen from the hyperplastic granulosa cells in the sex cords of this case is the most likely cause of the persistent estrus. Weekly vaginal cytologies for months confirm the presence of high levels of estrogen. Elevated estrogen is also a likely contributor to the rapid growth of the fibroleiomyoma. Only low levels of serum progesterone were present when measured. This case demonstrates the multisystemic effects of estrogen in a young bitch and the clinical presentation that was observed when abnormal ovarian development occurs.

## Concluding Remarks

This case is an unusual interaction of ovarian germ cell hypoplasia, hyperplastic granulosa cells producing estrogen, and a vaginal fibroleiomyoma in a young bitch. No other cases have been reported with this combination of abnormalities. The ovarian changes warrant classification as dysplasia instead of hypoplasia due to this unusual combination of changes.

The limitations of this case report include the unknown chromosome status of the patient and the unknown prevalence of these anomalies within the patient's littermates to evaluate for a genetic component. Future improvement could be made by staining the leiomyoma to confirm or deny the hypothesis that there are estrogen receptor-α's present that contributed to the growth of the leiomyoma.

## Data Availability Statement

The original contributions presented in the study are included in the article/supplementary material, further inquiries can be directed to the corresponding author.

## Author Contributions

SM contributed to writing the manuscript and literature review. DS and GS assessed the gross and histopathologic findings. JC contributed to critical revision of the manuscript, assisted with surgery on the patient, and managed the clinical case. All authors contributed to the final review.

## Conflict of Interest

The authors declare that the research was conducted in the absence of any commercial or financial relationships that could be construed as a potential conflict of interest.

## Publisher's Note

All claims expressed in this article are solely those of the authors and do not necessarily represent those of their affiliated organizations, or those of the publisher, the editors and the reviewers. Any product that may be evaluated in this article, or claim that may be made by its manufacturer, is not guaranteed or endorsed by the publisher.

## References

[1] MacLachlan NJ. Ovarian disorders in domestic animals. Environ Health Perspect. (1987) 73:27–33. 10.1289/ehp.877327 3665869 PMC1474553

[2] Millán Y Guil-Luna S Reymundo C Sánchez- Céspedes R Martín de las Mulas J. Sex steroid hormones and tumors in domestic animals. Insights Vet Med. (2013) 191–214. 10.5772/54324

[3] Millan Y Gordon A de los Monteros AE Reymundo C de las Mulas JM. Steroid receptors in canine and human female genital tract tumours with smooth muscle differentiation. J Comp Pathol. (2007) 136:197–201. 10.1016/j.jcpa.2007.01.004 17362977

[4] Goodman MH. Hormonal control of reproduction in the female. In: Basic Medical Endocrinology. 4th ed. Amsterdam, AM: Elsevier/Academic Press (2009). p. 257–75.

[5] Pitman JL McNeilly AS McNeilly JR Hays LE Bagby GC Jr. Sawyer HR. The fate of granulosa cells following premature oocyte loss and the development of ovarian cancers. Int J Dev Biol. (2012) 56:949–58. 10.1387/ijdb.120144jp 23417416

